# Arterial Spin Labeling Magnetic Resonance Imaging for Acute Disorders of Consciousness in the Intensive Care Unit

**DOI:** 10.1007/s12028-024-02031-0

**Published:** 2024-06-25

**Authors:** Elisabeth Waldemar Grønlund, Ulrich Lindberg, Patrick M. Fisher, Marwan H. Othman, Moshgan Amiri, Christine Sølling, Rune Damgaard Nielsen, Tenna Capion, Urszula Maria Ciochon, John Hauerberg, Sigurdur Thor Sigurdsson, Gerda Thomsen, Gitte Moos Knudsen, Jesper Kjaergaard, Vibeke Andrée Larsen, Kirsten Møller, Adam Espe Hansen, Daniel Kondziella

**Affiliations:** 1 Department of Neurology, Copenhagen University Hospital - Rigshospitalet, Blegdamsvej 9, 2100 Copenhagen, Denmark; 2 Functional Imaging Unit, Department of Clinical Physiology and Nuclear Medicine, Copenhagen University Hospital - Rigshospitalet, Glostrup, Denmark; 3 Neurobiology Research Unit, Copenhagen University Hospital - Rigshospitalet, Copenhagen, Denmark; 4 Department of Drug Design and Pharmacology, University of Copenhagen, Copenhagen, Denmark; 5 Department of Neuroanaesthesiology, Copenhagen University Hospital - Rigshospitalet, Copenhagen, Denmark; 6 Department of Neurosurgery, Copenhagen University Hospital - Rigshospitalet, Copenhagen, Denmark; 7 Department of Radiology, Copenhagen University Hospital - Rigshospitalet, Copenhagen, Denmark; 8 Department of Clinical Medicine, University of Copenhagen, Copenhagen, Denmark; 9 Department of Cardiology, Copenhagen University Hospital - Rigshospitalet, Copenhagen, Denmark

**Keywords:** Brain injury, Cerebral blood flow, Coma, Disorders of consciousness, Functional neuroimaging

## Abstract

**Background:**

To investigate patients with disorders of consciousness (DoC) for residual awareness, guidelines recommend quantifying glucose brain metabolism using positron emission tomography. However, this is not feasible in the intensive care unit (ICU). Cerebral blood flow (CBF) assessed by arterial spin labeling magnetic resonance imaging (ASL-MRI) could serve as a proxy for brain metabolism and reflect consciousness levels in acute DoC. We hypothesized that ASL-MRI would show compromised CBF in coma and unresponsive wakefulness states (UWS) but relatively preserved CBF in minimally conscious states (MCS) or better.

**Methods:**

We consecutively enrolled ICU patients with acute DoC and categorized them as being clinically unresponsive (i.e., coma or UWS [≤ UWS]) or low responsive (i.e., MCS or better [≥ MCS]). ASL-MRI was then acquired on 1.5 T or 3 T. Healthy controls were investigated with both 1.5 T and 3 T ASL-MRI.

**Results:**

We obtained 84 ASL-MRI scans from 59 participants, comprising 36 scans from 35 patients (11 women [31.4%]; median age 56 years, range 18–82 years; 24 ≤ UWS patients, 12 ≥ MCS patients; 32 nontraumatic brain injuries) and 48 scans from 24 healthy controls (12 women [50%]; median age 50 years, range 21–77 years). In linear mixed-effects models of whole-brain cortical CBF, patients had 16.2 mL/100 g/min lower CBF than healthy controls (*p* = 0.0041). However, ASL-MRI was unable to discriminate between ≤ UWS and ≥ MCS patients (whole-brain cortical CBF: *p* = 0.33; best hemisphere cortical CBF: *p* = 0.41). Numerical differences of regional CBF in the thalamus, amygdala, and brainstem in the two patient groups were statistically nonsignificant.

**Conclusions:**

CBF measurement in ICU patients using ASL-MRI is feasible but cannot distinguish between the lower and the upper ends of the acute DoC spectrum. We suggest that pilot testing of diagnostic interventions at the extremes of this spectrum is a time-efficient approach in the continued quest to develop DoC neuroimaging markers in the ICU.

**Supplementary Information:**

The online version contains supplementary material available at 10.1007/s12028-024-02031-0.

## Introduction

Coma affects 2 in 1,000 people each year [1], many of whom are admitted to the intensive care unit (ICU). A patient’s consciousness level has major implications for prognosis, treatment, resource allocation, and end-of-life decisions, but physicians are underestimating consciousness levels in up to 40% of unresponsive patients with brain injury [2, 3]. This is particularly worrisome in the ICU because underestimation of awareness levels may put patients at risk of suboptimal treatment decisions [4, 5]. Indeed, seven of ten deaths in the ICU occur because a good clinical outcome is deemed unlikely and life-sustaining therapy is withdrawn [4], so accurate estimation of preserved consciousness is crucial to avoid erroneous medical decisions, including premature withdrawal of life-sustaining therapy. Conversely, overestimation of awareness may lead to futile treatment, putting a strain on limited health care resources and causing caregiver distress. Therefore, precise estimation of consciousness levels after acute brain injury would lead to better prediction of clinical outcomes, optimization of neurorehabilitation potential, and a decrease in caregiver burden and health costs.

The European Academy of Neurology recommends 18-fluorodeoxyglucose positron emission tomography (FDG-PET) for the workup of unresponsive patients with chronic brain injury [3] because brain glucose metabolism is an excellent proxy for consciousness levels in these patients [6]. FDG-PET is known to be superior to functional magnetic resonance imaging (MRI) in detecting residual consciousness and prognosticating clinical outcomes in patients with chronic disorders of consciousness (DoC) [3, 6]. In patients with acute brain injury, however, FDG-PET is not feasible owing to logistical challenges in the ICU: There is rarely a clinical indication for an FDG-PET scan in ICU patients, and after administration of the radioactive tracer, there is a very limited time to perform the scan. Moreover, the scan duration of 30–60 min extends patient time outside the ICU, which increases the risk of complications. Hence, other neuroimaging measures than FDG-PET to assess consciousness levels in the ICU must be sought.

Arterial spin labeling magnetic resonance imaging (ASL-MRI) is a noninvasive method to quantify cerebral blood flow (CBF) using magnetically labeled arterial blood water as an endogenous tracer [7]. CBF is tightly coupled with glucose metabolism and neuronal activity, supporting ASL as a reliable measure of brain function [8]. ASL-MRI is validated against other established CBF measures [9–13], and ASL-MRI CBF measurements overlap considerably with FDG-PET measures in patients with dementia and epilepsy [14–18]. Notably, ASL-MRI can be performed in ICU patients in continuation of a clinically indicated structural MRI scan; the scan time is short (approximately 10 min), it is relatively inexpensive, and patients are not exposed to radioactivity [7, 16, 19].

In this proof-of-principle study, we hypothesized that CBF assessed by ASL-MRI can distinguish between clinically unresponsive and clinically low responsive ICU patients (and thus might be able to identify residual consciousness). Hence, we enrolled, on one hand, patients with DoC with known residual consciousness levels, in whom ASL-MRI should show relatively preserved CBF, and on the other hand, patients with clinically definite loss of consciousness, in whom ASL-MRI should reveal severely compromised CBF. In addition, we acquired ASL-MRI at both 1.5 T and 3 T in a sample of healthy volunteers to assess the reliability of CBF measurements across different magnetic field strengths, as some patients are contraindicated for 3 T scans. We reasoned that, if positive, this proof-of-principle study would lay the foundation for a larger prospective trial to investigate the usefulness of ASL-MRI to predict consciousness levels in ICU patients with acute DoC.

## Methods

### Patient Cohort

We enrolled a consecutive sample of mechanically ventilated patients with DoC admitted to the ICU of a tertiary referral center (Rigshospitalet, Copenhagen University Hospital) who fulfilled the following inclusion criteria: (1) minimum age > 18 years, (2) clinically nonresponding with an acute DoC after traumatic or nontraumatic brain injury, (3) < 31 days since injury, (4) clinical stability allowing transportation to an MRI scanner outside the ICU, and (5) written informed consent obtained from legal guardian. Exclusion criteria were (1) acute life-threatening conditions, (2) high-dose sedation, (3) clinically overt seizures or nonconvulsive seizures on (continuous) electroencephalogram (EEG), (4) a history of high-grade carotid artery stenosis (90%), (5) body temperature < 35 °C, and (6) contraindications to MRI.

We aimed for normoventilation and the lowest possible levels of sedation. If patients could not be fully weaned off sedation, dosages were reduced to the lowest possible levels as previously described [20, 21]. Levels of sedation were graded as (1) none or minimal, indicating absence of intravenous fentanyl, remifentanil, propofol, midazolam, sodium thiopental, or sevoflurane; (2) low to moderate, indicating fentanyl < 500 µg/h or < 200 µg/h if combined with propofol, remifentanil < 1000 µg/h or < 250 µg/h if combined with propofol, propofol < 100 mg/h, midazolam < 10 mg/h, or sevoflurane < 3%; or (3) high or very high, indicating propofol ≥ 100 mg/h, fentanyl ≥ 500 µg/h or ≥ 200 µg/h if combined with propofol, remifentanil ≥ 1,000 µg/h or ≥ 250 µg/h if combined with propofol, midazolam ≥ 10 mg/h, sevoflurane ≥ 3%, or any dosage of sodium thiopental [22].

Consciousness levels were determined by a clinical examination just prior to the scan on the same day by trained medical staff (EWG, MHO, and MA) under the supervision of a board-certified neurologist with > 15 years of experience in neurocritical care (DK). The neurological examination included the following: (1) assessment of cranial nerves and sensorimotor status, (2) Glasgow Coma Scale (GCS), (3) Full Outline of Unresponsiveness (FOUR) score [23], (4) Simplified Evaluation of Consciousness Disorders (SECONDs) [24], (5) visual pursuit/fixation with a mirror, (6) ability to follow simple motor commands (including with the family, when possible, to stimulate arousal), (7) reaction to central and peripheral noxious stimuli (in the absence of command-following), and (8) assessment of verbal and nonverbal communication, as described earlier [25] and in accordance with the European Academy of Neurology Guideline on Coma and other Disorders of Consciousness [3]. Patients were subclassified into the following categories: coma, unresponsive wakefulness state (UWS), minimally conscious state with/without language processing (MCS−/MCS+), or emerged from MCS. For the group analysis, patients were dichotomized into the following groups: those with residual consciousness (MCS or better [≥ MCS]) and those without residual consciousness (coma or UWS [≤ UWS]). In addition, we accessed patients’ electronic health records to identify relevant health information, including medical history, diagnoses, treatments, and laboratory and imaging test results.

### Healthy Controls

Healthy volunteers were recruited through a local database of potential research participants with an interest in participating in brain research. We included 24 healthy controls, two men and two women from each age decade ranging from 20 to 80 years without any of the following exclusion criteria: (1) major neurological or psychiatric disease, including (but not limited to) prior cognitive impairment; (2) antipsychotic medication; (3) systolic blood pressure > 200 mm Hg and diastolic blood pressure > 115 mm Hg; (4) average weekly alcohol consumption of > 14 and > 21 units for women and men, respectively; (5) use of euphoric drugs, including cannabis, within the preceding 3 months; (6) pregnant or breastfeeding; (7) previous participation in trials with radioactivity (with accumulated radiation doses of > 10 mSv) within the last year or significant work-related exposure to radioactivity; and (8) contraindications to MRI. Participants were screened for cognitive impairment using the Mini Mental Status Examination, and neurological deficits were ruled out by a standard clinical neurological examination. Blood pressure and routine blood tests were obtained. Healthy participants received 140 Danish kroner (US $20) per hour as economic compensation. All participants gave written informed consent.

### ASL-MRI

ASL-MRI was performed on 1.5 T (Signa Artist) or 3 T (Signa Premier) MRI scanners (GE Healthcare) with 21- or 48-channel head coils, respectively. Patients were not sedated or, if necessary, receiving sevoflurane or propofol at the lowest possible dosages to limit movement artifacts; they were mechanically normoventilated and monitored for sedation, ventilator rate, blood pressure, pulse, and blood oxygenation by experienced neuroanesthesiologists. Hemoglobin levels (which are known to influence ASL-MRI results [26–28]) were retrieved from same-day blood samples in accordance with clinical routine laboratory procedures.

ASL-MRI images were acquired twice using the GE product 3D pcASL sequence with a post-labeling delay (PLD) of 1.525 or 2.525 s. Parameters at 3 T were as follows: labeling duration = 1.45 s, 512 sampling points on eight spirals, field of view = 24 cm, reconstructed matrix = 128, repetition time (TR) = 4.810 s (PLD = 1.525 s) or 5.810 s (PLD = 2.525 s), echo time 52.8 ms, bandwidth 62.5 Hz/pixel, slice thickness = 4 mm, number of slices = 36, acquisition time = 4:32 min (PLD = 1.525 s) or 5:21 min (PLD = 2.525 s), and number of excitations = 3. Parameters at 1.5 T were unchanged, except TR = 4.816 s (PLD = 1.5 s) or 5.816 s (PLD = 2.5 s), echo time 10.7 ms, and acquisition time 4:19/5:08 min. Quantitative CBF images were calculated automatically on the MRI scanners by vendor-provided software. A high-resolution three-dimensional T1-weighted structural image was acquired using a sagittal, magnetization-prepared rapid gradient echo sequence (repetition time/echo time/inversion time = 7.7/3.2/450 ms at 3 T and 7.3/3.0/450 ms at 1.5 T, flip angle = 12°, in-plane acquired matrix 256 × 256, number of slices = 220, slice thickness = 0.9 mm, [field of view (FOV); 230 mm]. Gray and white matter segmentation was performed with the FreeSurfer image analysis suite version 7.1.0–1 (http://surfer.nmr.mgh.harvard.edu/). Briefly, high-resolution T1-weighted scans were analyzed in FreeSurfer with tissue type segmentation as well as parcellations of the cortical areas of the brain according to the Desikan-Killiany atlas. Registration of the ASL CBF maps was conducted in FreeSurfer using the boundary-based registration algorithm. The cortical segmentation and parcellations were then resliced into the native ASL space from which median CBF values were extracted. Because ICU patients often have intracranial devices or are connected to medical equipment that is only compatible with 1.5 T MRI, we acquired ASL-MRI in patients at either 1.5 T or 3 T. To determine the effect of MRI field strength on the measured CBF, we acquired both 1.5 T and 3 T ASL-MRI data (performed at the same day) in all healthy controls.

### Data Analyses

The proposed sample size was an empirical estimate based on clinical feasibility and the number of study participants in similar proof-of-principle research testing the clinical extremes on the spectrum of consciousness levels to assess the value of prognostication methods in brain injury [29, 30] as well as the number of volunteers in our database. As stated previously, whole-brain gray matter CBF, as well as regional CBF levels in the amygdala, thalamus, and brainstem, was derived based on the cortical gray matter segmentation from FreeSurfer software resliced to the ASL-MRI. Furthermore, because cortical metabolism of the most preserved hemisphere correlates better with residual consciousness in chronic DoC than the whole-brain average [31], we also did a subanalysis based on CBF measurements in the best hemisphere of each patient.

With data from healthy controls, we report the replicability of CBF estimates across field strengths (1.5 T vs. 3 T) and PLDs (1.525 and 2.525 s) using Person’s *ρ* correlation coefficients and population intraclass correlation coefficients (ICC) based on a random effects model. Statistical analysis of CBF of patients and healthy controls employed linear mixed-effects modeling with study participant as a random effect and hemoglobin level as a fixed effect. Group was also modeled as a fixed effect, either with two levels (healthy controls vs. patients) or three levels (healthy control, ≤ UWS, and ≥ MCS, using either healthy control or ≥ MCS as reference). Results were considered significant at *p* < 0.05. Owing to the exploratory nature of the investigation, we did not correct for multiple testing. All analyses were done using MATLAB (version 2021a; The MathWorks Inc., Natick, MA).

### Ethics and Data Handling

Written informed consent was obtained from all participants or their next-of-kin. All procedures followed the Declaration of Helsinki. The study received ethical permission from the regional ethical committee (VEK Region Hovedstaden; H-21015473). All personal data were handled and stored according to the General Data Protection Regulations and Act, after approval of the local data protection authorities (P-2021-288).

## Results

**Fig. 1:**
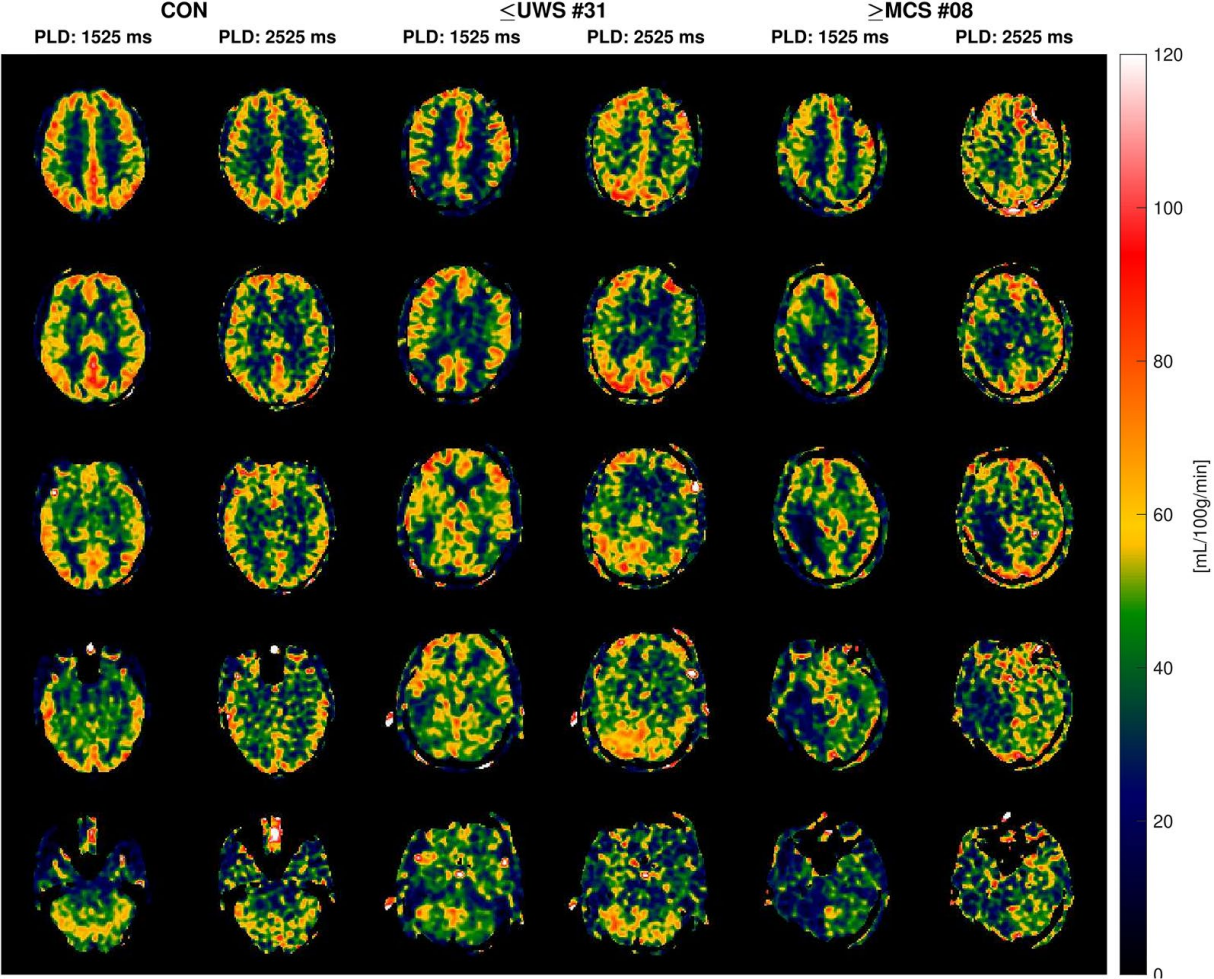
Arterial spin labeling magnetic resonance imaging examples of a healthy volunteer (CON), a patient in a coma (≤ UWS), and a minimally conscious state patient with command-following abilities (≥ MCS). Examples are study participants with median whole-brain gray matter cerebral blood flow in their groups. Images were acquired at 1.5 T field strength with post-labeling delays (PLDs) of 1.525 and 2.525 s; 10 equidistant slices are shown for each study participant

Between December 2021 and April 2023, we obtained 84 ASL-MRI scans from 59 participants: 35 ICU patients (32 nontraumatic brain injuries; 11 women; median age 56 years, range 18–82 years) who were investigated with a total of 36 ASL-MRI scans (at scan time: 24 times ≤ UWS, 12 times ≥ MCS, i.e., one patient was scanned twice) and 48 scans from 24 healthy volunteers (12 women [50%]; median age 50 years, range 21–77 years; i.e., healthy volunteers were investigated twice, at 1.5 T and 3 T). The primary cause of ICU admission was ischemic stroke in 13 (37.1%) patients, anoxic-ischemic encephalopathy in 4 (11.4%) patients, traumatic brain injury in 3 (8.6%) patients, intracerebral hemorrhage in 3 (8.6%) patients, neuroinfections in 3 (8.6%) patients, subarachnoid hemorrhage in 2 (5.7%) patients, and other etiologies in 7 (20.0%) patients. On average, ≤ UWS patients were scanned slightly earlier after their brain injury than ≥ MCS patients, but the difference was not statistically significant (7.9 ± 6.1 days vs. 10.5 ± 6.9 days, *p* = 0.27). Table 1 provides baseline characteristics of the patient cohort. Figure 1 provides representative ASL-MRI scans of a patient in a coma, an MCS + patient, and a healthy volunteer.

**Table 1:** Baseline characteristics of the patient cohort with disorders of consciousness

Scan ID	Sex	Age	Brain injury	Consciousness level^a^	Timing of ASL-MRI scan, days after injury	Sedation^b^	Hemoglobin (mmol/liter)	3-month outcome
1	M	56	Brainstem infarction (vertebrobasilar dolichoectasia)	Coma	3	None or minimal	9.0	Dead
2	M	25	Global anoxia, epileptic seizure, hydrocephalus	UWS	9	None or minimal	6.0	Dead
3	F	78	Multiple embolic strokes, hypertensive encephalopathy, sepsis	Coma	2	None or minimal	7.1	Dead
4	M	40	Posterior fossa tumor (ependymoma)	eMCS	3	Low to moderate	5.7	Alive
5	F	22	Cardiac arrest (strangulation)	UWS	19	Low to moderate	6.2	Alive
6	M	75	Thalamic hemorrhage (hypertensive)	Coma	18	Low to moderate	6.1	Alive
7	M	32	Hyponatremia (polydipsia)	MCS+	6	Low to moderate	9.1	Alive
8	F	55	Posterior fossa epidermoid cyst with postsurgical hemorrhage	MCS−	25	None or minimal	5.7	Alive
9	M	75	Brainstem infarction (basilar artery thrombosis)	Coma	1	High or very high	8.7	Dead
10	M	71	Subarachnoid hemorrhage with cardiac arrest	Coma	7	Low to moderate	7.6	Dead
11	M	72	Multifactorial encephalopathy (NPH, strokes, epilepsy)	MCS+	6	Low to moderate	7.7	Alive
12^c^	See ID 11	72	See ID 11	MCS+	14	Low to moderate	7.4	See ID 11
13	F	56	Aorta dissection, multiple cerebral emboli	MCS+	6	Low to moderate	6.2	Alive
14	M	82	Brainstem infarction (basilar artery thrombosis)	Coma	2	Low to moderate	6.8	Dead
15	M	67	Aorta dissection, multiple cerebral emboli	Coma	4	Low to moderate	6.6	Dead
16	F	44	Pontine hemorrhage (hypertensive)	Coma	5	High or very high	7.3	Alive
17	F	59	Hepatic/uremic encephalopathy, rhabdomyolysis, seizures	Coma	10	Low to moderate	5.1	Alive
18	F	52	Traumatic brain injury, epilepsy	UWS	6	High or very high	6.1	Alive
19	M	55	Posterior circulation stroke (basilar artery thrombosis)	UWS	5	Low to moderate	5.7	Dead
20	M	51	Pontine hemorrhage (hypertensive)	MCS−	11	Low to moderate	7.3	Alive
21	M	23	Cardiac arrest (strangulation)	UWS	15	Low to moderate	6.8	Alive
22	M	40	Cerebellar infarct with fossa posterior decompression	MCS+	13	None or minimal	5.7	Alive
23	M	78	Posterior circulation stroke, status epilepticus	Coma	3	Low to moderate	12.4	Alive
24	F	76	Pneumococcal meningitis	Coma	10	Low to moderate	5.0	Dead
25	M	50	Acute leukoencephalopathy, COVID-19	Coma	5	None or minimal	9.0	Dead
26	M	73	Multiple embolic strokes	MCS−	3	Low to moderate	9.3	Dead
27	M	58	Posterior circulation stroke (basilar artery thrombosis)	Coma	1	None or minimal	9.3	Dead
28	M	67	Third ventricle tumor	UWS	21	Low to moderate	4.8	Dead
29	M	30	Traumatic brain injury	Coma	15	High or very high	5.4	Alive
30	M	48	Subarachnoid hemorrhage	Coma	19	High or very high	5.7	Alive
31	F	18	Traumatic brain injury	Coma	7	Low to moderate	6.3	Alive
32	F	48	Astrocytoma, postsurgical abscess	MCS−	24	Low to moderate	4.9	Dead
33	M	77	Posterior cerebellar stroke	MCS−	5	Low to moderate	8.6	Alive
34	M	52	Left middle cerebral artery infarction	MCS−	10	Low to moderate	5.7	Dead
35	F	61	Myocardial infarction, hypotension	UWS	10	Low to moderate	9.8	Alive
36	M	75	Herpes simplex encephalitis	UWS	9	Low to moderate	5.9	Alive

*ASL-MRI* arterial spin labeling magnetic resonance imaging, *eMCS* emerged from minimally conscious state, *F* female, *M* male, *MCS*+ minimally conscious state with command-following, *MCS*− minimally conscious state without command-following, *NPH* normal pressure hydrocephalus, *UWS* unresponsive wakefulness state

^a^For group analysis, patients were dichotomized into coma and UWS (≤ UWS) and MCS or better (≥ MCS)

^b^See Methods for details

^c^This patient was investigated twice with 9 days in-between the two scans

### CBF at Different Field Strengths and PLDs in Healthy Controls

**Fig. 2:**
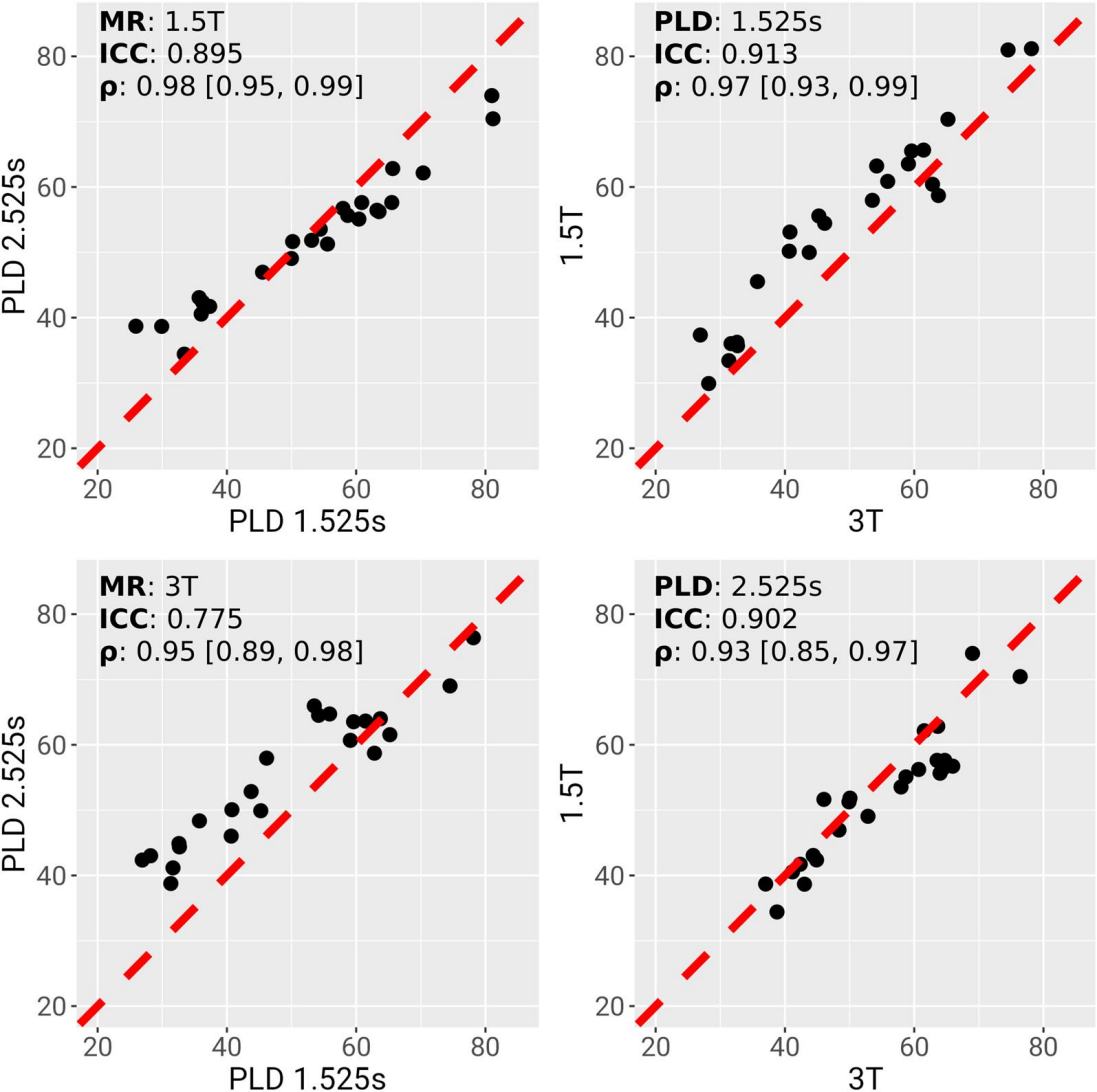
Replicability of cerebral blood flow (CBF) estimates across post-labeling delays (PLDs) and magnetic resonance (MR) field strengths. Four plots show the correspondence between global gray-matter-weighted CBF estimates acquired on one of two MR scanners (3 T or 1.5 T) and at one of two PLDs (1.525 or 2.525 s). Top left, two PLDs acquired on 1.5 T scanner; top right, two MR strengths at PLD 1.525 s; bottom left, two PLDs acquired on 3 T scanner; bottom right, two MR strengths at PLD 2.525 s. *x*- and *y*-axes are in units of CBF. Black dots represent individual participants. Red hatched lines show the identity line. ρ denotes Pearson’s correlation coefficient and associated 95% confidence interval. ICC intraclass correlation coefficient

In healthy controls, CBF ASL-MRI showed a strong correlation across field strengths of 1.5 T and 3 T, irrespective of PLD (PLD 1.525 s: correlation coefficient = 0.97 [95% confidence interval (CI) 0.93–0.99], ICC = 0.91; PLD 2.525 s: correlation coefficient = 0.93 [95% CI 0.85–0.97], ICC = 0.90; Fig. 2). Similarly, CBF was strongly correlated across PLDs (1.5 T: correlation coefficient = 0.98 [95% CI 0.95–0.99], ICC = 0.90; 3 T: correlation coefficient = 0.95 [95% CI 0.89–0.98], ICC = 0.78; Fig. 2). Visually, the image quality of the PLD 1.525 s ASL images was better, and those images were chosen for subsequent statistical analyses.

### CBF in ICU Patients with DoC Compared with Healthy Controls

**Fig. 3:**
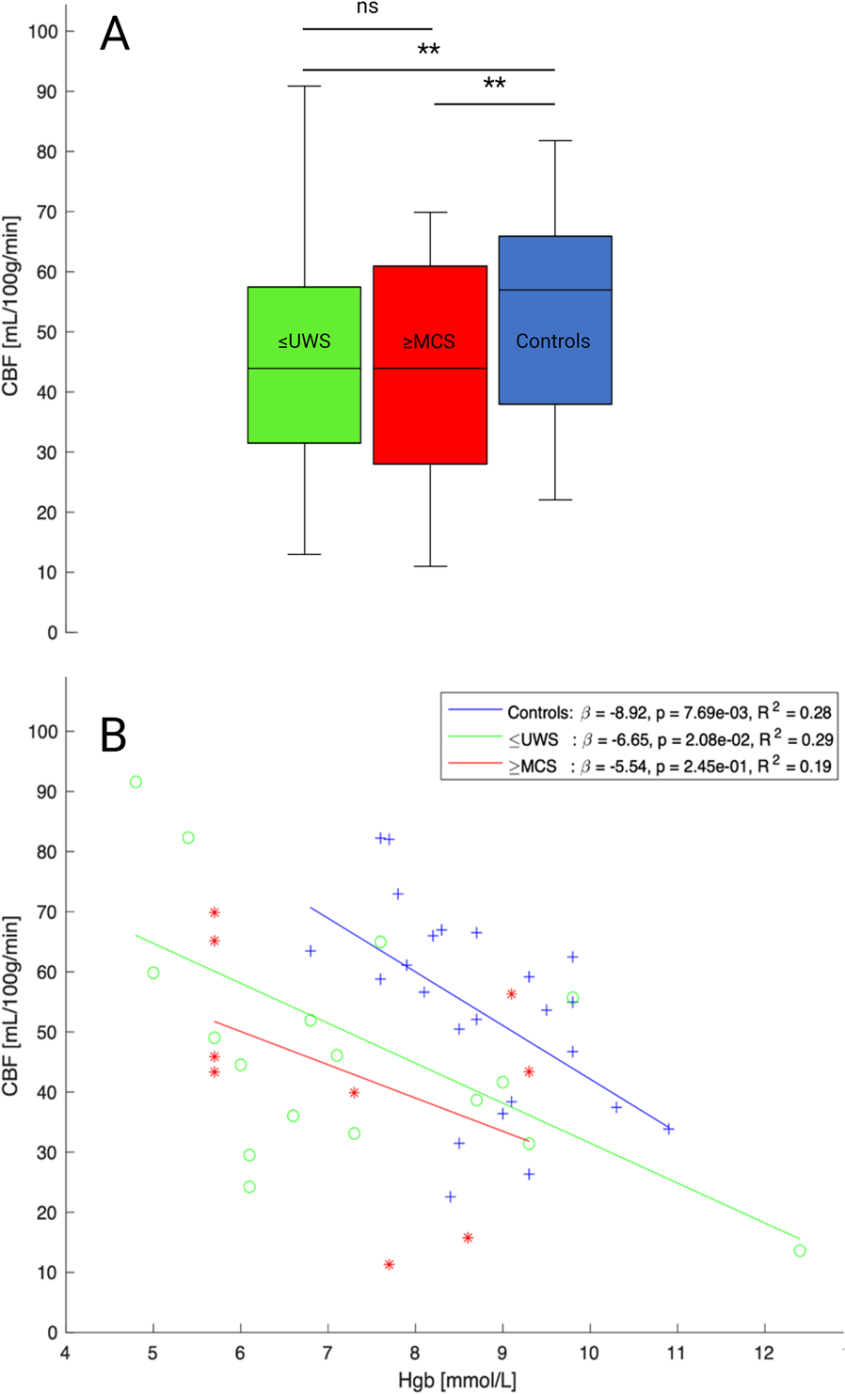
**a** Box and whisker plot showing cerebral blood flow (CBF) levels measured at 1.5 T arterial spin labeling magnetic resonance imaging in coma or unresponsive wakefulness state (≤ UWS) and minimally conscious state or better (≥ MCS) patients and healthy controls. ns, not significant. ** *p* < 0.005. **b** CBF of the three groups as a function of hemoglobin levels. Although statistically significant only in controls (*p* = 0.001), all groups showed decreasing CBF with increasing hemoglobin levels

Group comparisons between patients and healthy controls are presented here for data from 1.5 T scans and PLD 2.525 s. In the linear mixed-effects model comparing healthy controls and patients, ASL-MRI whole-brain cortical CBF values showed a significant group difference (*p* = 0.0041; Fig. 3, Table 2). Patients had on average 16.2 mL/100 g/min lower CBF compared with healthy controls. Hemoglobin was significantly negatively correlated with CBF in the linear mixed-effects model (*p* = 0.0001; Table 2). A post hoc linear regression analysis within groups showed that hemoglobin levels were significantly negatively correlated with CBF for healthy controls (*p* = 0.007, *R*^2^ = 0.28) and ≤ UWS patients (*p* = 0.02, *R*^2^ = 0.29), whereas a nonsignificant negative correlation was observed for ≥ MCS patients (*p* = 0.25, *R*^2^ = 0.19; Fig. 3). Group comparisons with patient data from both 1.5 T and 3 T did not reveal statistically different results (data not shown).

**Table 2:** Multiple regression results from linear mixed-effects models

	Reference group	Region	CBF difference, mL/100 g/min, (95% CI)	Effect of Hgb, mL/100 g/min/(mmol/L)
≤ UWS plus ≥ MCS^a^	Controls	Cortex (mean)	− 16.2 (− 26.9 to − 5.4), ***p* = 0.0041**	− 6.92, ***p* = 0.0001**
≤ UWS^b^	Controls	Cortex (mean)	− 13.9 (− 25.6 to − 2.3), ***p* = 0.02**	− 6.91, ***p* = 0.0001**
≥ MCS^b^	Controls	Cortex (mean)	− 20.6 (− 34.7 to − 6.5), ***p* = 0.005**	
≤ UWS^c^	≥ MCS	Cortex (mean)	6.7 (− 7.1 to 20.5), *p* = 0.33	
≤ UWS^c^	≥ MCS	Cortex (median)	8.3 (− 6.6 to 23.1), *p* = 0.27	− 6.72, ***p* = 0.0002**
≤ UWS^c^	≥ MCS	Thalamus	6.0 (− 7.1 to 19.1), *p* = 0.36	− 6.02, ***p* = 0.0002**
≤ UWS^c^	≥ MCS	Amygdala	3.9 (− 7.1 to 14.9), *p* = 0.48	− 4.34, ***p* = 0.001**
≤ UWS^c^	≥ MCS	Brainstem	3.5 (− 8.0 to 15.0), *p* = 0.54	− 4.19, ***p* = 0.0025**

Bold *p* values are statistically significant (see Methods)

CBF, cerebral blood flow, CI, confidence interval, Hgb, hemoglobin, MCS, minimally conscious state with or without command-following, UWS, unresponsive wakefulness state

^a^Combined patient groups (i.e., unresponsive [≤ UWS] and low responsive [≥ MCS] ICU patients) versus healthy controls

^b^Separate patient groups of ≤ UWS and ≥ MCS patients versus healthy controls

^c^ ≤ UWS patients versus ≥ MCS patients

### Whole-Brain, Hemispheric, and Regional CBF in ICU Patients with DoC Stratified According to Consciousness Levels

In the linear mixed-effects model, compared with controls, the whole-brain CBF was 13.9 mL/100 g/min lower in ≤ UWS patients (*p* = 0.02) and 20.6 mL/100 g/min lower in ≥ MCS patients (*p* = 0.005; Table 2). However, there was no statistically significant difference in ASL-MRI scans between consciousness levels (i.e., ≤ UWS vs. ≥ MCS) in patients (*p* = 0.33; Table 2). In a subanalysis of hemispheric CBF, absolute differences between the best and worst brain hemispheres in patients were overall small (Supplemental Fig. S1), and best hemispheric CBF did not differ between patient groups (*p* = 0.41; Supplemental Fig. S2). Numerical differences between ≤ UWS and ≥ MCS patients regarding regional CBF in the thalamus, amygdala, and brainstem were statistically nonsignificant. For all inspected brain areas, hemoglobin levels were negatively correlated with CBF (Table 2).

## Discussion

We investigated whether CBF measured by ASL-MRI could provide information about residual awareness in ICU patients with acute DoC. However, although ASL-MRI was feasible in this logistically challenging patient population, CBF values could not discriminate between ≤ UWS and ≥ MCS patients.

### CBF is Decreased in Acute DoC

CBF in ICU patients with DoC was overall reduced compared with that in healthy controls, with 16 mL per 100 g brain tissue per minute on average. However, despite reduced global CBF at the group level, we found a striking interindividual difference in CBF both in ≤ UWS and ≥ MCS patients. The range of CBF values in patients with DoC (from around 10 to 90 mL per 100 g per minute) exceeded that of healthy controls on both sides of the spectrum (around 20–80 mL per 100 g per minute), consistent with a 15–20% CBF variation between young healthy study participants [32]. This might reflect a combination of disturbed cerebral autoregulation in acute brain injury [33] and iatrogenic interventions with inotropic drugs to increase the cerebral perfusion pressure [34]. Other possible factors include different levels of partial pressure of carbon dioxide (PaCO_2_), body temperature, and sedation levels (even though the latter were kept to a minimum). However, this CBF variability may reveal an opportunity to study trajectories of CBF over time (as opposed to isolated CBF measurements) as markers for declining or improving consciousness in acute brain injury. Given that hemoglobin levels are correlated with CBF [35] and influence ASL-MRI results [26–28], such trajectory studies would need to account for daily hemoglobin values.

Although CBF is well studied in neurological conditions such as epilepsy and dementia, few studies have investigated it in patients with DoC [36–39]. Using whole-brain computed tomography brain perfusion, Xiong et al. [36] examined the relationship between consciousness level on one side and CBF, cerebral blood volume, and time to peak on the other side in 29 patients with chronic UWS and 47 with chronic MCS. UWS patients had significantly decreased CBF in bilateral frontal, temporal, and occipital lobes, as well as in the thalamus and the brainstem. GCS and FOUR scores were positively correlated with CBF, cerebral blood volume, and time to peak in almost all regions of interest. However, this study differed from ours by use of another neuroimaging modality (i.e., computed tomography brain perfusion), the setting (i.e., prolonged DoC in chronic brain injury as opposed to ICU patients with acute brain injury), and the lack of a control group.

### ASL-MRI Cannot Discriminate Between Consciousness Levels in Acute DoC

After adjustment for hemoglobin levels, CBF was decreased in patients compared with healthy controls; however, global CBF was not significantly different between patient groups. Regional CBF levels in the cortex, thalamus, amygdala, or brainstem were also not statistically different in ≤ UWS patients compared with ≥ MCS patients, and neither was best hemispheric CBF.

Less than a handful of studies, including ours, have investigated CBF in DoC using ASL-MRI [37–39]. Except for possibly one [38], none of these studies showed meaningful differences between patients with and without residual consciousness. In a retrospective pilot study of 12 ICU patients with aneurysmal subarachnoid hemorrhage, Nelson et al. [37] analyzed CBF within gray matter nodes of the default mode network (DMN), which include bilateral medial prefrontal cortices, thalami, and posterior cingulate cortices, yet not all these patients had a DoC. There were no correlations between CBF and admission GCS scores for any DMN nodes, perhaps because of the small sample size, ASL data acquisition at variable times after the brain injury, and the possibility that CBF analysis in DMN nodes may not reflect the functional integrity of the entire neural network. In an even smaller study of four study participants who met MCS criteria [39], CBF was decreased in gray matter compared with that in normal controls (*n* = 10), especially in the medial prefrontal and midfrontal regions. Although CBF patterns showed considerable variability (from 7.7 to 33.1 mL/100 g/min), in the one study participant who was studied longitudinally, global CBF values increased over time and correlated with clinical improvement [39]. In yet another study (*n* = 23 patients), Wu et al. [38] found that regional CBF in MCS patients was increased in the putamen, anterior cingulate gyrus, and medial frontal cortex compared with that in UWS patients. A difference between MCS and UWS in a left-lateralized pattern was observed, but the authors did not probe predictive utility. Unlike our study, these were patients with DoC in the postacute to chronic setting with a median time from brain injury onset of 3 months (range 1–47 months). It might therefore be that contrary to the acute phase, ASL-MRI correlates better with consciousness levels in the chronic stage when reactive cerebral inflammatory processes and dysregulation of cerebral autoregulation have resolved. Ideally, this hypothesis should be tested in longitudinal follow-up studies with repeated ASL-MRI measurements in the acute and chronic stages of brain injury.

### Strengths and Limitations

Our work has limitations. First, patients were scanned at either 1.5 T or 3 T MRI depending on clinical availability and contraindications. This reflects real-world challenges inherent to clinical neuroimaging of ICU patients. Nonetheless, our data from healthy volunteers (who were scanned twice, on 1.5 T and 3 T) indicate that ASL-MRI was highly replicable across magnetic field strengths. Second, our patient cohort was somewhat skewed toward nontraumatic brain injuries, possibly reflecting the greater clinical need for high-resolution imaging in these etiologies, but we believe it is unlikely that a more balanced patient cohort would have yielded substantially different results. Third, except for hemoglobin levels, we did not correct our statistical models for other physiological markers, such as PaCO_2_, body temperature, and sedation levels, which might be worthwhile to investigate in replication studies. Moreover, we did not correct for focal brain lesions, but visual inspection of ASL CBF maps and brain tissue segmentation revealed no evidence that they were negatively affected by focal lesions. Also, because we used the median value of the entire cortex, we do not assume that focal lesions or infarcts had a large influence on the results. In fact, we did a subanalysis by calculating CBF for the best hemisphere only and found no difference between unresponsive and low responsive patients. In future studies, one might consider adjusting for volumetric measurements when analyzing smaller regions such as the brainstem or subcortical gray matter to decrease partial volume effects from adjacent low-signal regions. Finally, we might have identified a discriminative signal of ASL-MRI had we included a larger patient cohort. However, if we have missed such a signal, it cannot be clinically useful given that such an effect size would be small. We therefore conclude that the number of patients was sufficient for our overarching goal, which was to decide whether a large multicenter trial to test the diagnostic accuracy of a single ASL-MRI study early after brain injury is warranted (the answer is no).

We think the present work also has strengths: It involves a larger patient cohort than previous studies, and, to our knowledge, it is the first prospective ASL-MRI study in patients with acute DoC in the ICU with an equally large control group. Furthermore, we showed that ASL-MRI is feasible in acute DoC and that CBF measurements are reliable across different field strengths and PLDs, possibly supporting broader clinical implementation for serial CBF measurements. Finally, as stated previously, we fulfilled our primary objective: Before embarking on resource-intensive diagnostic phase 2b trials in the ICU [20], we suggest that feasibility studies like ours that test diagnostic accuracy of interventions at the extremes of the DoC spectrum are valuable for go/no-go decisions, depending on the apparent potential of the diagnostic procedure in question. We have previously used a similar approach based on relatively small DoC ICU cohorts investigated with measurements of otoacoustic emissions [29], mental arithmetic [30], neurovascular coupling [40], and brimonidine eye drops [41] to probe for clinical outcome predictions and residual consciousness in this challenging patient population.

## Conclusions

ASL-MRI is feasible in the ICU and (at least in healthy controls) reliable across different magnetic field strengths and PLDs. CBF measurements can distinguish well between ICU patients with brain injury and healthy controls but lack discriminatory value to identify the absence or presence of residual consciousness in acute DoC. Intraindividual CBF variability and CBF trajectories over time may yield greater insights than cross-sectional ASL-MRI measurements, which may be an approach worth further investigation. Testing novel diagnostic interventions at the extremes of the DoC spectrum might help to tell promising interventions apart from less promising ones. This design appears to be a resource-sensitive and time-efficient approach to continue the quest for refined neuroimaging technologies to detect residual consciousness after acute brain injury in the ICU.

## Supplementary Information

Below is the link to the electronic supplementary material.

Supplementary file1 (PDF 114 kb)

## Data Availability

Anonymous raw data and code will be shared upon reasonable request. Structural MRI and ASL-MRI scans cannot be anonymized and will not be shared, but radiological reports of both structural MRI and ASL-MRI scans are available on request.
