# Supplementary material for: Arterial Spin Labeling Magnetic Resonance Imaging for Acute Disorders of Consciousness in the Intensive Care Unit
Source: Neurocrit Care. 2024 Jun 25;41(3):1027–37. doi: 10.1007/s12028-024-02031-0 (PMC11599417; doi:10.1007/s12028-024-02031-0)
Supplement: Supplementary file 1 — Supplementary file1 (PDF 114 kb) [file 12028_2024_2031_MOESM1_ESM.pdf]

Supplemental file: Grønlund et al. Arterial spin labeling MRI for acute disorders of consciousness in the ICU

Figure S1: Box and whisker plot showing CBF levels of the best (“max hemi”) and worst (“min hemi”) hemispheres all DoC patients measured at 1.5T ALS-MRI.

Figure S2: Best hemispheric CBF of ≤UWS and ≥MCS patients and of healthy controls (right hemisphere) as a function of hemoglobin levels. All groups showed decreasing CBF with increasing hemoglobin levels.

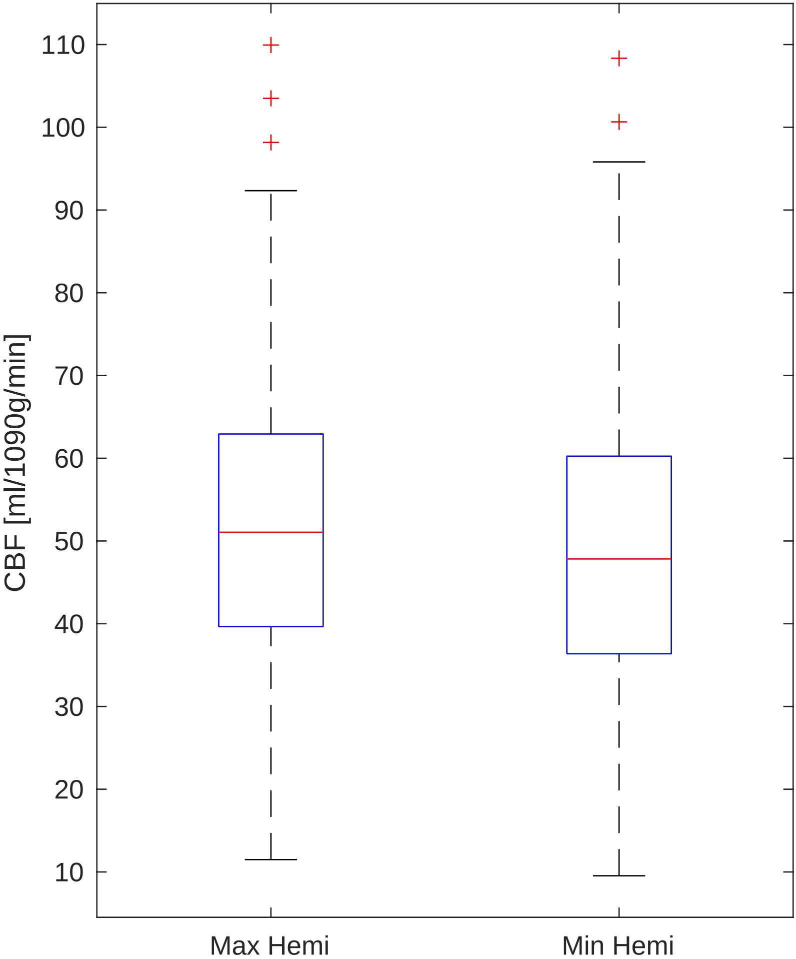
CBF [ml/1090g/min]
110
100
90
80
70
60
50
40
30
20
10
Max Hemi
Min Hemi

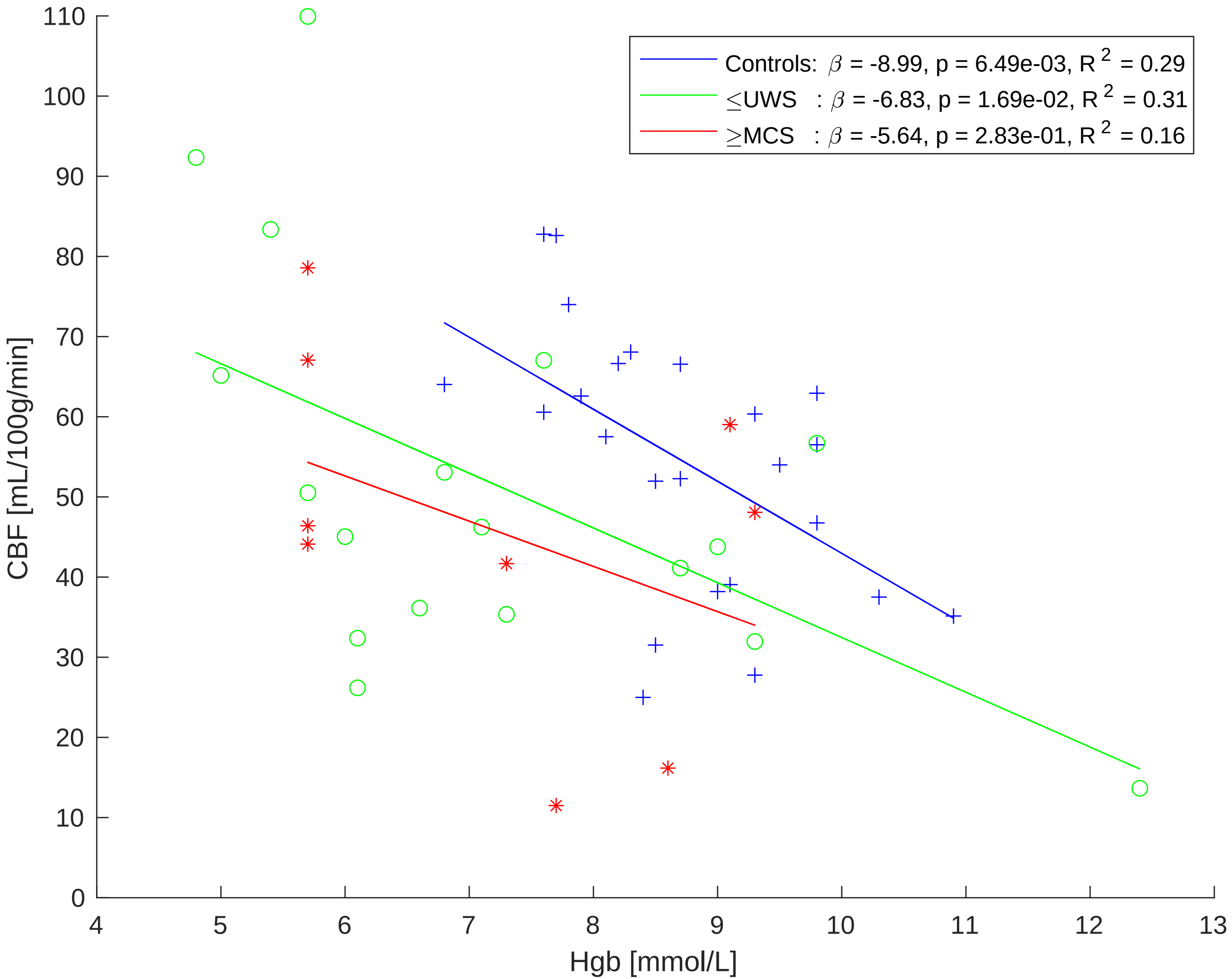

Controls: β = -8.99, p = 6.49e-03, R 2 = 0.29
≤UWS : β = -6.83, p = 1.69e-02, R 2 = 0.31
≥MCS : β = -5.64, p = 2.83e-01, R 2 = 0.16
CBF [mL/100g/min]
Hgb [mmol/L]
0
10
20
30
40
50
60
70
80
90
100
110
4
5
6
7
8
9
10
11
12
13
